# Structurally engineered colloidal quantum dot phosphor using TiO_2_ photonic crystal backbone

**DOI:** 10.1038/s41377-022-01020-2

**Published:** 2022-11-01

**Authors:** Hansol Lee, Tae-Yun Lee, Yeonsang Park, Kyung-Sang Cho, Young-Geun Rho, Hyuck Choo, Heonsu Jeon

**Affiliations:** 1 Department of Physics and Astronomy, Seoul National University, Seoul, 08826 Republic of Korea; 2 Inter-university Semiconductor Research Center, Seoul National University, Seoul, 08826 Republic of Korea; 3 Department of Physics, Chungnam National University, Daejeon, 34134 Republic of Korea; 4 Institute of Quantum Systems, Chungnam National University, Daejeon, 34134 Republic of Korea; 5 Samsung Advanced Institute of Technology, Suwon, 16678 Republic of Korea; 6 Institute of Applied Physics, Seoul National University, Seoul, 08826 Republic of Korea

**Keywords:** Photonic crystals, Green photonics

## Abstract

Photonic crystal (PhC) phosphor, in which the phosphor material is periodically modulated for an enhancement in color-conversion efficiency via resonant absorption of excitation photons, is a paradigm-shifting structural phosphor platform. Two-dimensional (2D) square-lattice PhC phosphor is currently considered the most advanced platform because of not only its high efficiency, but also its immunity to excitation polarization. In the present study, two major modifications are made to further improve the performance of the 2D PhC phosphor: increasing the refractive index contrast and planarizing the surface. The index contrast is improved by replacing the PhC backbone material with TiO_2_ whereas the surface planarization is achieved by removing excessive colloidal quantum dots from the surface. In comparison with the reference phosphor, the upgraded PhC phosphor exhibits ~59 times enhanced absorption (in simulations) and ~7 times enhanced emission (in experiments), both of which are unprecedentedly high. Our results not only brighten the viability and applicability of the PhC phosphor but also spur the phosphor development through structural engineering of phosphor materials.

## Abstract (web summary)

Two-dimensional photonic crystal phosphor is upgraded in terms of both material and structure, which results in ~7 times enhanced color conversion efficiency compared with reference phosphor.

## Graphical abstract

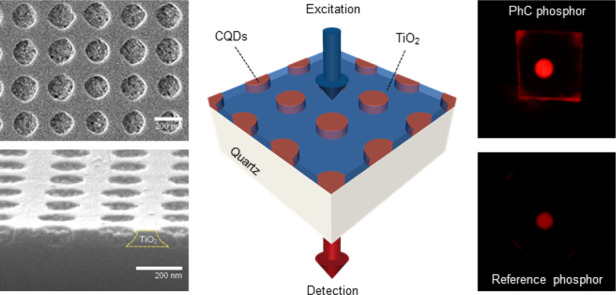

## Introduction

Phosphor provides a compact and efficient light source that is pivotal in modern display and solid-state lighting technologies, when combined with a short-wavelength light-emitting diode (LED)^1–5^. The main stream of phosphor development, however, still remains traditional and materials-oriented. Recently, it has been noted that phosphor efficiency can be considerably improved via structural engineering based on the principles of nanophotonics^6–9^. In particular, the authors proposed a concept to shape phosphor material into a planar thin-film photonic crystal (PhC) structure with its Γ-point band-edge mode tuned to the energy for phosphor excitation^9^. The PhC phosphor engineered in this manner resonates with excitation photons that are incident perpendicular to the phosphor plane; note that the in-plane momentum of the vertically incident photon is zero (*k*_||_ = 0) that corresponds to the Γ-point (or Brillouin zone center) in the reciprocal momentum space. The resonant excitation results in a significant enhancement^10–13^ in the fluorescence from phosphor material via the enhanced absorption of excitation photons, thereby affording higher phosphor efficiency.

The most advanced PhC phosphor platform demonstrated until now employs a two-dimensional (2D) square-lattice PhC slab waveguide structure^9^. In addition to an improved phosphor efficiency, the square-lattice PhC structure offers a 90° rotational symmetry that effectively removes the polarization dependence of the excitation photons^14–17^; thus, it is suitable for an unpolarized excitation source such as an LED. Nonetheless, there is still scope for further improvement in the 2D PhC phosphor. In our previous studies^6,7,9^, Si_3_N_4_ (*n*_SiN_ ≈ 2.05) has been the high-index material used in the PhC backbone structure, while densely packed colloidal quantum dots (CQDs; *n*_CQD_ ≈ 1.83) serve as both the phosphor and the low-index material. The combination of Si_3_N_4_ and CQDs offers the index contrast of only *n*_SiN_/*n*_CQD_ ≈ 1.12, thus limiting the strength of the resultant PhC effects (Supplementary Information S1).

Pertaining to phosphor materials, CQDs are becoming more popular than ever, rapidly replacing the conventional host-activator type of phosphor materials represented mostly by YAG:Ce^3+^. In fact, consumer electronic appliances based on CQD-based display components are ubiquitous. This is because CQDs have many unique advantages, such as simple wet-chemical synthesis (still monodisperse in size), convenient emission color control (by quantum confinement effect), high internal quantum efficiency (through surface passivation), and broad absorption bandwidth (owing to continuous density of states)^18–20^. In addition, CQDs are completely compatible with the PhC phosphor structure; CQDs are only a few nanometers in size^21–23^, whereas the critical dimensions of the PhC phosphor are of the order of 100 nm. Spin-coating has been the most convenient and reliable method for applying CQDs in the PhC backbone structure. However, it inevitably results in a partially conformal surface morphology^6,9,24–26^; such a nonplanar wavy surface broadens the linewidth of the Γ-point resonance, thus degrading the Q-factor of the resonance mode (Supplementary Information S2).

Based on the above discussions, in this study, two modifications were made to the 2D PhC phosphor, one in the material and the other in the structure. First, we switched the PhC backbone material from Si_3_N_4_ to TiO_2_ (*n*_TiO_ ≈ 2.61), thereby increasing the index contrast to *n*_TiO_/*n*_CQD_ ≈ 1.42. Secondly, we removed the excessive CQDs from the PhC surface via the so-called ‘squeegee’ method^27,28^ to obtain an overall planar PhC phosphor structure. The successful implementation of these two modifications results in the enhancement factors of ~59 in the absorption of the excitation photons (confirmed by simulations) and ~7 in the CQD fluorescence (measured in experiments) over the reference phosphor that is unstructured, but contains an equal amount of CQDs. While the significant discrepancy between the simulations and experiments indicates massive scope for further improvement, the experimentally determined fluorescence enhancement factor is thus far the largest value realized.

## Results

### Comparisons among different PhC phosphor platforms

**Fig. 1:**
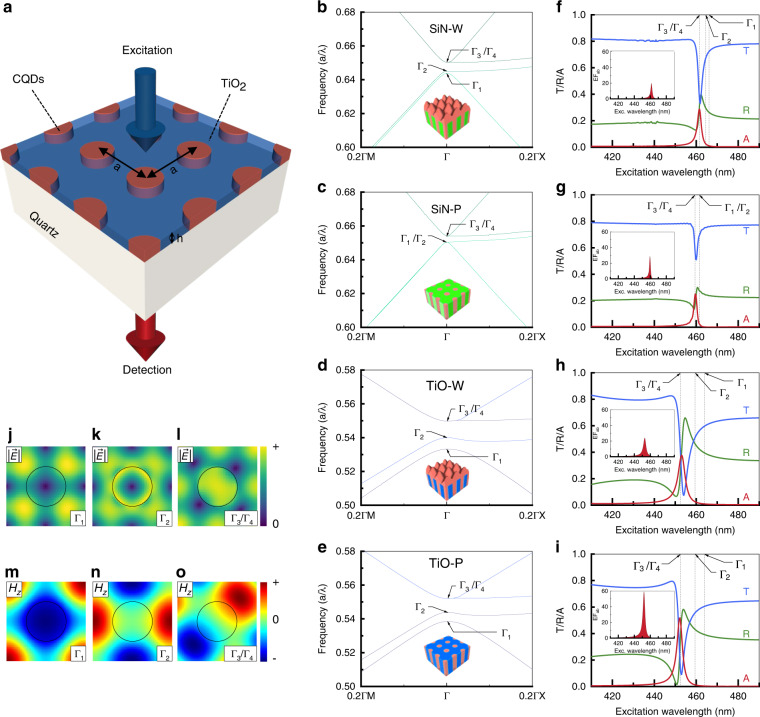
CQD-based PhC phosphor with a TiO2 2D PhC backbone layer. **a** Schematic of the 2D PhC phosphor structure, with the schemes for excitation and detection indicated. **b**–**e** Photonic band structures calculated for **b** SiN-W, **c** SiN-P, **d** TiO-W, and **e** TiO-P. The inset in each figure is a schematic of the corresponding PhC phosphor platform. Both the Si_3_N_4_ and TiO_2_ films are assumed to be 60 nm in thickness. **f**–**i** Calculated transmittance, reflectance, and absorbance spectra for **f** SiN-W, **g** SiN-P, **h** TiO-W, and **i** TiO-P. Insets show the calculated absorption enhancement factors for the corresponding PhC phosphor platforms. For direct comparisons, the wavelengths of the Γ-point band-edge modes determined from the band structures in (**b**–**e**) are identified. **j**–**l** Γ-point band-edge mode profiles ($\left| E \right| = \sqrt {E_x^2 + E_y^2 + E_z^2}$) calculated for TiO-P: **j** Γ_1_, **k** Γ_2_, and **l** Γ_3_/Γ_4_. **m**–**o** Γ-point band-edge mode profiles (*H*_z_) calculated for TiO-P: **m** Γ_1_, **n** Γ_2_, and **o** Γ_3_/Γ_4_. The circles in (**j**–**o**) represent the air hole in the PhC unit cell

Figure 1a schematically depicts the 2D PhC phosphor structure proposed in this study. A square-lattice TiO_2_ PhC backbone slab consisting of an array of air holes filled with CQDs is formed on the fused quartz substrate. With a blue LED in mind as a convenient and compact excitation source, the PhC phosphor is designed to be excited at *λ*_exc_ ≈ 450 nm. Excitation photons are assumed to be incident from the top (phosphor side), while CQD fluorescence is observed at the bottom (substrate side). The present PhC phosphor structure differs from the previous structure^9^ in two aspects; the material that constitutes the PhC backbone is TiO_2_, rather than Si_3_N_4_, and the CQDs are leveled, such that the entire surface becomes planar.

Calculations were performed to make systematic and comparative evaluations of various PhC phosphor platforms. Figure 1b–e shows the band structures calculated for the four different PhC phosphor platforms: Si_3_N_4_ backbone with a wavy CQD surface (SiN-W), Si_3_N_4_ backbone with the planar CQD surface (SiN-P), TiO_2_ backbone with a wavy CQD surface (TiO-W), and TiO_2_ backbone with the planar CQD surface (TiO-P). The most distinct change induced by replacing the backbone material from Si_3_N_4_ to TiO_2_ (that is, increased index contrast) is the opening and widening of the photonic bandgap^29,30^. Consequently, more pronounced PhC effects are expected^31–35^. To address the impacts of the modifications quantitatively, we calculated the reflectance (R), transmittance (T), and absorbance (A = 1 − R − T) of the four PhC phosphor platforms; the results are shown in Fig. 1f–i. All the PhC phosphor platforms commonly exhibit a single resonance peak at the wavelength of the degenerate Γ_3_/Γ_4_ mode identified in the corresponding band structure. In fact, we are particularly interested in the air band mode above the bandgap because its electric field modal profile significantly overlaps with the air holes where CQDs are present. Figure 1j–l compares the electric field profiles $\left( {\left| E \right| = \sqrt {E_x^2 + E_y^2 + E_z^2} } \right)$ of the Γ_1_, Γ_2_, and Γ_3_/Γ_4_ modes of TiO-P, respectively. Although Fig. 1f–i suggests that the resonance effects are most pronounced for TiO-P with the maximum absorbance exceeding A = 0.5, direct comparisons in terms of the peak absorbance are inadequate because the amounts of CQDs contained in the PhC phosphor platforms are not equal.

We deduced the absorption enhancement factor (*EF*_*ab*_) that is the ratio of the absorbance of the excitation photons by the PhC phosphor to that by the reference phosphor. The reference phosphor is a thin and homogeneous film containing an equal amount of CQDs to that in the corresponding PhC phosphor. The results are shown in the insets of Fig. 1f–i. TiO-P indeed outperforms the others in terms of the maximum absorption enhancement factor $\left( {{\mathrm{EF}}_{{\mathrm{ab}}}^{{\mathrm{max}}}} \right)$. Nonetheless, another useful parameter for performance assessment is the resonance width because phosphors are typically excited by a broad bandwidth source. An appropriate figure-of-merit can accordingly be the weighted enhancement factor defined by ${\mathrm{WEF}}_{{\mathrm{ab}}} = {\int} {{\mathrm{EF}}_{{\mathrm{ab}}}(\lambda ) \cdot G_{{\mathrm{exc}}}(\lambda )d\lambda }$, where the normalized Gaussian function $G_{{\mathrm{exc}}}(\lambda )$ represents the power spectrum of an hypothetical excitation light source with the center wavelength tuned at the resonance peak and the linewidth set for 30 nm in the full width at half maximum (FWHM). All the parameter values extracted from Fig. 1f–i are listed in Table 1. On one hand, in terms of ${\mathrm{EF}}_{{\mathrm{ab}}}^{{\mathrm{max}}}$, the performance improves in the order of SiN-W < TiO-W < SiN-P«TiO-P, the highest value being ${\mathrm{EF}}_{{\mathrm{ab}}}^{{\mathrm{max}}} \approx 59$ for TiO-P. Assessing by *WEF*_*ab*_, on the other hand, the performance order changes to SiN-W < SiN-P«TiO-W«TiO-P. Regardless of the comparison standards, we can therefore conclude that TiO-P is the best platform.

**Table 1:** The maximum absorption enhancement factors and the weighted absorption enhancement factors calculated for the four PhC phosphor platforms

PhC phosphor	${\mathrm{EF}}_{{\mathrm{ab}}}^{{\mathrm{max}}}$	${\mathrm{WEF}}_{{\mathrm{ab}}}$
SiN-W	19.6	2.4
SiN-P	28.6	2.8
TiO-W	24.1	4.6
TiO-P	59.2	8.7

Although the PhC phosphor is designed for the Γ-point band-edges (or for the vertically incident excitation photons), the resonant absorption can still occur at other band points (or for oblique excitation). For clarification, the absorbance spectra are calculated for a few different incidence angles and their correspondence with the photonic band structure is confirmed (Supplementary Information S3). It is also worth noting that Fig. 1f–i does not show any sign of resonant absorption at Γ_1_ and Γ_2_. This is rather surprising, even if their intrinsic modal mismatch with the CQD-filled air holes is considered; note that Γ_1_ and Γ_2_ belong to the dielectric bands. However, the Γ_1_ and Γ_2_ band-edge modes of the square-lattice 2D PhC slab are known to be the bound states in the continuum (BIC) with diverging Q-factors, inferring that their coupling to the plane waves in free space is strictly forbidden by the structural symmetry^36–40^. Figure 1m–o shows the magnetic field profiles (*H*_z_) of the Γ_1_, Γ_2_, and Γ_3_/Γ_4_ modes of TiO-P, respectively, in which the *C*_2_ symmetry under 180° rotation, the sufficient condition for forming a BIC, exists for Γ_1_ and Γ_2_, not for Γ_3_/Γ_4_.

### Fabrication of the PhC phosphor

**Fig. 2:**
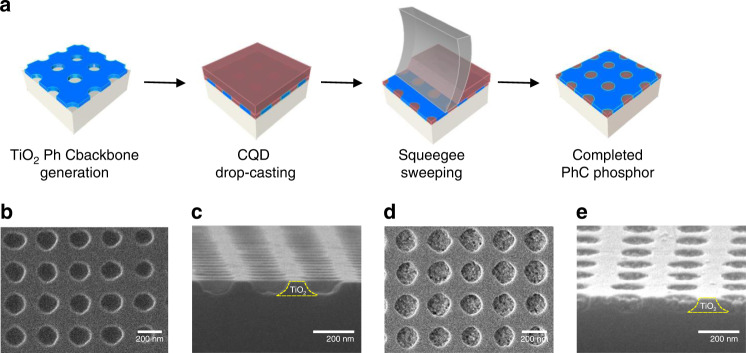
Fabrication of the CQD-based 2D PhC phosphor. **a** Process flow depicted by the representative processing steps: 2D PhC pattern generation in the TiO_2_ backbone layer, drop-casting of CQDs on top of the 2D PhC backbone structure, squeegee sweeping for the surface planarization, and the completed PhC phosphor. **b**–**e** SEM images of the 2D PhC phosphor: **b**, **c** before and **d**, **e** after the selective CQD incorporation into the air holes

Based on the calculation results, the TiO-P PhC phosphor platform was realized. The fabrication sequence is outlined schematically in Fig. 2a. The first step was the deposition of a 60 nm-thick TiO_2_ backbone layer by sputtering. Thereafter, a 2D PhC pattern with a square-lattice array of air holes (lattice constant *a* = 250 nm; air-hole radius *r*/*a* = 0.25) was generated by applying two-beam laser interference lithography (LIL) twice. The LIL is a maskless photolithographic method used to generate submicron-period grating patterns at a high throughput over a large area, whereby the grating period can be conveniently adjusted by the angle of the incident laser beams^41,42^. Inductively-coupled-plasma reactive-ion etch (ICP-RIE) was followed to transfer the PhC pattern down to the underlying TiO_2_ layer. Red-emitting CdSe-CdS-ZnS core-shell-shell CQDs diluted in cyclohexane solution was drop-casted on the TiO_2_ PhC backbone structure, after which a squeegee was swept across the sample surface to remove excessive CQDs. The reference phosphor was also prepared carefully by spin-coating the CQDs directly on a planar quartz plate, ensuring the composition of the same amount of CQDs per unit area as the PhC phosphor. Further details of the phosphor preparations can be found in Methods section. Scanning electron microscope (SEM) images were acquired before (Fig. 2b, c) and after (Fig. 2d, e) the CQD deposition. Although the final outcome is reasonably close to the intended structure, a few structural non-idealities are identified from the SEM images. For example, the shape and size of the air holes fluctuate (Fig. 2b, d), while the TiO_2_ etch sidewalls are not vertical, but slanted (~73°) (Fig. 2c, e). These are attributed to the poor pattern definition by the LIL, the lack of hard etch mask, and the non-optimized ICP-RIE process. In addition, the squeegee process was not accomplished as intended. Although the TiO_2_ surface is free of CQDs, the CQDs in the air holes are partially removed, such that the CQD profiles are slightly dented into the air holes, as seen in Fig. 2e. These structural imperfections adversely impact the PhC phosphor performance, which will be discussed later.

### Photoluminescence excitation measurements

**Fig. 3:**
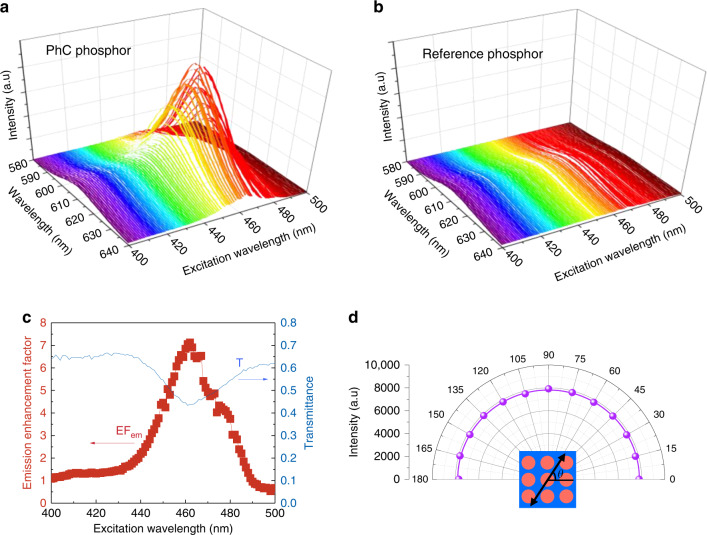
Enhanced CQD fluorescence of the 2D PhC phosphor. **a**, **b** Measured CQD fluorescence spectra for **a** the 2D PhC phosphor and **b** the reference phosphor, both measured as a function of the excitation wavelength. **c** Emission enhancement factor plotted as a function of the excitation wavelength. Also shown for comparison is the measured transmittance spectra. **d** CQD fluorescence intensity at the resonance wavelength, measured as a function of the polarization angle of excitation photons. The line is for reference. The inset is for identifying the polarization angle with respect to the square-lattice PhC pattern

Photoluminescence excitation (PLE) experiments were performed to characterize the fabricated PhC phosphor. A wavelength-selectable excitation source with a wide scan range was custom built by combining a Xe lamp and a monochromator. Figure 3a, b shows the CQD fluorescence spectra from the PhC phosphor and the reference phosphor, respectively, measured at various excitation wavelengths. The CQD fluorescence intensity from the PhC phosphor is resonantly enhanced near the excitation wavelength of *λ*_exc_ ≈ 460 nm, whereas that from the reference phosphor does not react at all to the excitation wavelength. It is worth noting that it is only the fluorescence intensity that changes dramatically, whereas the intrinsic spectral shape of the CQD fluorescence is preserved, regardless of the phosphor type and excitation wavelength.

The performance of the fabricated TiO-P PhC phosphor can be expressed by the emission enhancement factor (*EF*_*em*_), defined as the CQD fluorescence intensity ratio of the PhC phosphor to the reference. Figure 3c shows the emission enhancement factor deduced from Fig. 3a, b that is characterized by a broad resonance with the peak enhancement factor of ${\mathrm{EF}}_{{\mathrm{em}}}^{{\mathrm{max}}} \approx 7.1$. Although this value is substantially smaller than the theoretically obtained absorption enhancement factor $\left( {{\mathrm{EF}}_{{\mathrm{ab}}}^{{\mathrm{max}}} \approx 59} \right)$, it is still the largest enhancement factor ever reported for the PhC phosphor (and also for any structurally engineered phosphor, to the best of our knowledge). The large discrepancy between the calculations and experiments is attributed mainly to the structural imperfections induced during actual device fabrication (as mentioned earlier, Fig. 2b–e). Such structural imperfections can result in a reduction in the peak enhancement factor and also an inhomogeneous broadening of resonance (Supplementary Information S4). However, the resonance broadening is not necessarily disadvantageous because it still allows efficient absorption over a wider spectral range that is particularly desirable when the light source for phosphor excitation has a broad emission bandwidth. In this context, the integrated emission enhancement factor, ${\mathrm{IEF}}_{{\mathrm{em}}} = {\int}_{{\mathrm{EF}} > 1} {{\mathrm{EF}}_{{\mathrm{em}}}\left( \lambda \right)d\lambda \approx {\mathrm{EF}}_{{\mathrm{em}}}^{{\mathrm{max}}} \times \Delta \lambda _{{\mathrm{em}}}}$, should be a useful parameter to quantify the PhC phosphor performance. The effective linewidth Δλ_*em*_ determined from Fig. 3c is 39 nm, comparable to the emission linewidth of a typical GaN-based LED. We also measured the transmittance spectrum of the fabricated PhC phosphor that exhibits a dip, as shown in Fig. 3c. The spectral position and width of the transmittance dip match with those of the resonance peak in the emission enhancement factor spectrum, indicating that the enhanced CQD fluorescence is indeed a result of the PhC structure.

As mentioned previously, phosphor excitation is typically achieved by an unpolarized light source. The use of the square-lattice PhC structure is therefore preferred because the 90° rotational symmetry of the square-lattice PhC eliminates the polarization dependence of the excitation photons effectively and efficiently^9^. For confirmation, we measured the CQD fluorescence intensity from the TiO-P PhC phosphor as a function of the polarization angle of excitation photons at the resonance wavelength. As shown in Fig. 3d, the CQD fluorescence intensity is completely insensitive to the excitation polarization, proving that our 2D square-lattice PhC phosphor is ideal for an unpolarized excitation source.

### Imaging of PhC phosphor performance

**Fig. 4:**
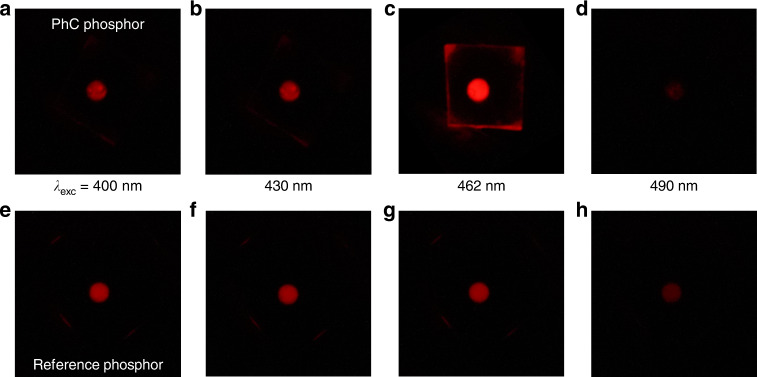
Visual comparisons of CQD fluorescence. Acquired photographs of **a**–**d** the 2D PhC phosphor and **e**–**h** the reference phosphor upon excitation at specific representative wavelengths: **a**, **e** *λ*_exc_ = 400 nm, **b**, **f** 430 nm, **c**, **g** 462 nm (resonance), and **d**, **h** 490 nm. All the images were acquired with a long-pass filter inserted to cut off the excitation photons. The sample size, which is outlined in (**c**), is ~1 × 1 cm^2^

A visual comparison between the fluorescence of the PhC and reference phosphors should be the most direct and decisive approach toward demonstrating the superiority of the PhC phosphor. Figure 4 shows the photographs for both the PhC (Fig. 4a–d) and reference (Fig. 4e–h) phosphors, acquired at four representative excitation wavelengths across the resonance: *λ*_exc_ = 400, 430, 462 (resonance), and 490 nm. Under the off-resonant excitation conditions, no apparent enhancement in favor of the PhC phosphor is observed. We notice that the CQD fluorescence at *λ*_exc_ = 490 nm is considerably weaker than those at *λ*_exc_ = 400 nm and 430 nm for both phosphors that is mainly due to the intrinsic dispersion of the CQD film—the longer the wavelength, the smaller the extinction coefficient (Supplementary Information S5). We also notice that at *λ*_exc_ = 490 nm the CQD fluorescence from the PhC phosphor is even weaker than that from the reference. This observation is consistent with Fig. 3c, where the fluorescence enhancement factor becomes less than 1 for *λ*_exc_ > 485 nm. Our speculation is that the Fano-type asymmetric resonance at the Γ-point band-edge modes causes a fast decay in the longer wavelength side, resulting in such inferior CQD fluorescence from the PhC phosphor. In drastic contrast, when the excitation wavelength is tuned to the resonance at *λ*_exc_ = 462 nm, the CQD fluorescence from the PhC phosphor far exceeds that from the reference. The red CQD fluorescence is extremely intense, such that even the fluorescence guided through the quartz substrate and scattered off at the quartz substrate edges is clearly visible (Fig. 4c).

## Discussion

Two upgrades were adopted to improve the performance of the 2D PhC phosphor—the employment of the high-index material TiO_2_ for the PhC backbone structure and the planarization of an otherwise wavy CQD surface. The former enhances the refractive index contrast of the PhC structure and the resultant PhC effects, while the latter reduces the scattering loss of the excitation photons, sharpening the Γ-point resonance. Both the changes strengthen the interaction between the excitation photons and phosphor material at the resonance wavelength and therefore, should render an improvement in the CQD fluorescence. The CQD fluorescence from a fabricated 2D PhC phosphor was measured to be enhanced ~7 times over the reference phosphor. Comparisons of the experimental results with simulation results indicate that there is still massive scope for further improvement by refining the fabrication processes, such as the LIL for homogeneous hole patterns and the ICP-RIE for vertical sidewalls.

## Materials and methods

### Device fabrication

A 60-nm-thick TiO_2_ film was rf-sputter-deposited on a fused quartz substrate using a TiO_2_ target in O_2_ environment at room temperature. A 2D square-lattice PhC pattern was generated by applying two-beam laser interference lithography twice; the sample was rotated by 90° after the first exposure. The PhC pattern was subsequently transferred to the underlying TiO_2_ layer via inductively-coupled-plasma reactive-ion etching (FABStar, TTL) in a gas mixture HBr/BCl_3_/Ar (5:20:40 in sccm). CdSe-CdS-ZnS core-shell-shell CQDs dispersed in cyclohexane solution in 0.4 wt% were drop-casted on the 2D PhC TiO_2_ backbone structure that was immediately followed by squeegee, sweeping to remove excessive CQDs on the sample surface. The squeegee was prepared by cutting a 5 mm-thick polydimethylsiloxane plate into a 1 × 2 cm^2^ piece. For comparison, a reference phosphor structure was also prepared by spin-coating the CQDs dispersed in cyclohexane solution of 0.2 wt% at 4000 rpm on another quartz substrate, resulting in a ~12 nm-thick planar CQD film containing the CQD amount per unit area equivalent to that in the PhC phosphor.

### Optical measurements

A wavelength-tunable excitation source with a wide tuning range was developed by combining a Xe lamp (6271 Xenon Arc Lamp, Newport) and a monochromator (CM110, Spectral Products) to investigate the CQD fluorescence spectrum as a function of the excitation wavelength. The width of the monochromator slit was adjusted, such that the spectral linewidth (or measurement resolution) became ~2 nm in the FWHM. The CQD fluorescence spectra were measured in transmission geometry, in which the excitation beam was directly incident on the phosphor surface, while the fluorescence spectra were acquired from the other side through the fused quartz substrate using a spectrometer (Kymera 193i-A Spectrometer with iVac 316 CCD, ANDOR). A polarizer was inserted in front of the phosphor sample to control the polarization angle of the vertically incident excitation photons. For transmittance spectrum measurement, a halogen illuminator (FOK-100W, Fiber Optic Korea) was used as a white light source, while the transmitted signal was fed into a visible/near-infrared fiber-optic spectrometer (HR4000CG-UV-NIR, Ocean Optics) through a bundled fiber input port.

### FDTD simulations

All numerical simulations were performed using a commercial software package (FDTD Solutions, Lumerical Solutions), and the results were subsequently analyzed to extract photonic band structures and absorbance/transmittance spectra. The dispersion relations of the refractive indices (both real and imaginary parts) for the TiO_2_ and CQD films were obtained from separate spectroscopic ellipsometry measurements.

## Supplementary information

Supplementary Information
