# Supplementary material for: Structurally engineered colloidal quantum dot phosphor using TiO2 photonic crystal backbone
Source: Light Sci Appl. 2022 Nov 1;11:318. doi: 10.1038/s41377-022-01020-2 (PMC9626542; doi:10.1038/s41377-022-01020-2)
Supplement: Supplementary file 1 — Supplementary Information [file 41377_2022_1020_MOESM1_ESM.docx]

Supplementary Information

**S1. Effects of the refractive index contrast**

**S2. Effects of the CQD surface morphology**

**S3. Incidence angle dependence**

**S4. Effects of the fluctuation in air-hole sizes**

**S5. Optical dispersions of the CQD film**

**S1. Effects of the refractive index contrast**

The width of photonic bandgap is calculated for the planar PhC phosphor platform as a function of the refractive index of the high-index material; the low-index material is fixed to a CQD film (*n*_CQD_ ≈ 1.83). As the refractive index of the high-index material increases⎯starting from that of Si_3_N_4_ (*n*_SiN_ ≈ 2.05) and going beyond that of TiO_2_ (*n*_TiO_ ≈ 2.61), the bandgap width increases monotonically, and thus the band structure effects are strengthened.

Quartz

n_H_

CQD

**Fig. S1**. Photonic bandgap width versus the refractive index of the high-index material, calculated using FDTD simulation. The red line is a linear fit to the simulation data. The inset schematically shows the calculation model structure for the planar PhC phosphor.

**S2. Effects of the CQD surface morphology**

The Q-factor of the degenerate Γ_3_/Γ_4_ mode is calculated as a function of the step height between the dielectric and air-hole regions. The total amount of CQDs is kept the same as that of the planar PhC structure. To facilitate the calculations, the model structure is simplified to have a rectangular symmetry. It is clearly shown that the Q-factor of the Γ_3_/Γ_4_ mode decreases monotonically as the step height increases.

h

Quartz

TiO_2_

CQD

**Fig. S2**. Q-factor of the Γ_3_/Γ_4_ mode as a function of the step height. The inset is a schematic of the calculation model structure.

**S3. Incidence angle dependence**

The PhC phosphor is designed and implemented based on the Γ-point band-edge mode, i.e., for vertically incident photons. However, the absorption resonance can occur for obliquely incident excitation photons as well. To demonstrate the resonant absorption for the oblique incidence and its correlation with the photonic band structure, the absorbance spectra for the TiO-P PhC phosphor are calculated at a few representative incidence angles (θ, ϕ). The resonance frequencies and wavevectors deduced from the calculated absorbance peaks are plotted in the PhC band structure.

Θ=0

Θ=30°,Ф=0

Θ=45°,Ф=0

Θ=0°,Ф=45°

Θ=30°,Ф=45°

Θ=15°,Ф=45°

Θ=15°,Ф=0

**Fig. S3**. Photonic band structure for the TiO-P PhC phosphor. The resonance peaks observed in the absorbance spectra calculated for different incidence angles (θ, ϕ) are identified in the band structure.

**S4. Effects of the fluctuation in air-hole sizes**

FDTD simulations are performed to examine the effects of the inhomogeneous air holes on the performance of the PhC phosphor. The air-hole sizes are assumed to vary randomly over the range of *a*/3 ≤ *d* ≤ 2*a*/3, where *d* and *a* are the air-hole diameter and the PhC lattice constant, respectively. To simplify the model structure and also to reflect the nature of the laser interference lithography, the circularity in the individual air-hole shape and the periodicity in the air-hole arrangement are kept. Shown below are the calculated absorbance spectra of the ideal and realistic PhC phosphor structures. Compared with the ideal one, the realistic PhC phosphor exhibits a significantly lower resonance peak with a broader linewidth. The simulations are repeated for more realistic model structures with different air-hole configurations, but the results are very similar.

**Fig. S4**. Calculated absorbance spectra for the ideal and realistic PhC phosphor structures.

**S5. Optical dispersions of the CQD film**

The complex refractive index (*n* − *ik*) of the CQD film was measured by spectroscopic ellipsometry technique, using an independently prepared CQD film. In particular, the dispersion in *k* across 400 nm < λ < 500 nm indicates that the absorption in this wavelength range varies wildly.

**Fig. S5**. Wavelength dispersions in the complex refractive index (*n* − *ik*) of the CQD film. The CQD film was prepared from the same batch of the CQD solution as the one used for the actual phosphor samples.
